# Loss of calcium/calmodulin-dependent protein kinase kinase 2, transferrin, and transferrin receptor proteins in the temporal cortex of Alzheimer’s patients postmortem is associated with abnormal iron homeostasis: implications for patient survival

**DOI:** 10.3389/fcell.2024.1469751

**Published:** 2024-11-28

**Authors:** Mohammad Golam Sabbir

**Affiliations:** ^1^ Department of Psychology and Neuroscience, College of Psychology, Nova Southeastern University, Fort Lauderdale, FL, United States; ^2^ Alzo Biosciences Inc., SanDiego, CA, United States

**Keywords:** Alzheimer’s disease, calcium, Ca^2+^/calmodulin (CaM)-dependent protein kinase kinase 2, homeostasis, iron, Parkinson’s disease, transferrin, transferrin receptor

## Abstract

**Introduction:**

Iron is crucial for brain function, but excessive iron is neurotoxic. Abnormally high brain iron accumulation is one of the pathogenic factors in Alzheimer’s disease (AD). Therefore, understanding the mechanistic basis of iron dyshomeostasis in AD is vital for disease mitigation. Calcium, another essential bioelement involved in cell signaling, also exhibits dysregulated homeostasis in AD. Calcium ion (Ca^2+^) signaling can influence iron homeostasis through multiple effectors. Our previous studies identified Ca^2+^/calmodulin (CAM)-dependent protein kinase kinase 2 (CAMKK2) as a regulator of transferrin (TF)-bound iron trafficking through the TF receptor (TFRC). Given CAMKK2’s high expression in brain cells, it was hypothesized that abnormal CAMKK2-TF/TFRC signaling may underlie excessive iron deposition in AD brains. This study aims to retrospectively investigate CAMKK2, TF, TFRC proteins, and iron content in temporal cortex tissues from AD patients and cognitively normal (CN) individuals, postmortem.

**Methods:**

Postmortem temporal cortex tissues from 74 AD patients, 27 Parkinson’s disease (PD) patients, and 17 CN individuals were analyzed for CAMKK2, TF, and TFRC protein levels by Western blotting. Additionally, prefrontal/temporal cortex tissues from 30 CN individuals of various ages were examined for age-related effects. Iron content in cortical tissues was measured using a colorimetric assay.

**Results:**

CAMKK2, TF, and TFRC levels were significantly decreased in AD patients’ temporal cortices compared to CN individuals, independent of age or postmortem interval-related changes. PD patients’ also exhibited similar reductions in CAMKK2/TF/TFRC levels. The increased iron content in AD brains was significantly correlated with reduced TF/TFRC protein levels.

**Discussion:**

Building on the previous identification of CAMKK2 as a regulator of TF/TFRC trafficking and iron homeostasis, the findings from this study suggest that downregulation of CAMKK2 in AD cortices may disrupt TF/TFRC signaling and contribute to iron overloading and neurodegeneration through iron-induced toxicity. The decreased levels of TF/TFRC and increased iron in AD brains may result from enhanced clearance or post-trafficking degradation of TF/TFRC due to CAMKK2 downregulation. Restoring CAMKK2 levels in the AD brain could offer a novel therapeutic approach to reestablish iron homeostasis. Further studies are needed to explore the pathways linking CAMKK2 and iron dysregulation in AD and other neurodegenerative diseases.

## 1 Introduction

Iron is an essential element for the development and functionality of the human brain. Its ability to exist in multiple oxidation states, primarily ferrous (Fe^2^⁺) and ferric (Fe³⁺), enables it to participate in various redox reactions and form stable complexes with other biomolecules such as oxygen, nitrogen, and sulfur atoms (Sánchez et al., 2017). This versatility, combined with its efficiency in electron carrying, makes iron indispensable in a wide range of biological processes, from energy production and oxygen transport to DNA synthesis and metabolic regulation (Abbaspour et al., 2014). However, the same properties that make iron useful also render it potentially toxic, as iron is a strong promoter of reactive oxygen species (ROS) that can drive the oxidation of proteins, lipid peroxidation, and nucleic acid modifications, ultimately compromising vital cellular functions and leading to cell death (Gutteridge and Halliwell, 2018). Consequently, anomalies in iron distribution and concentration in different tissues are associated with various diseases, including neurodegenerative diseases such as Alzheimer’s disease (AD), in which abnormal iron deposition in the brain has been identified as a pathogenic factor (Liu et al., 2018; Levi et al., 2024; Bartzokis et al., 2007).

The iron level generally increases in the aging brain (Bartzokis et al., 1997), but in AD, there is a dramatic increase in brain iron content (Altamura and Muckenthaler, 2009). Magnetic resonance imaging (MRI) has shown higher iron concentrations in the deep gray matter and neocortical regions in AD patients (N = 100) compared to cognitively normal (CN) individuals (N = 100) (Damulina et al., 2020). Iron binds to amyloid beta (Aβ) and influences its toxicity in the central nervous system (Joppe et al., 2019; Butterfield and Kanski, 2001). Studies have demonstrated that iron accumulates in the same brain regions in AD patients characterized by hallmark Aβ deposition, such as the hippocampus, parietal cortex, and motor cortex (Good et al., 1992; Dedman et al., 1992; Zecca et al., 2004), suggesting a connection between iron dysregulation and AD pathology. However, whether iron is a primary actor in the neurodegenerative process in AD or its alteration is a consequence of disease progression remains an open question.

Unlike most organs, the brain is protected by a vascular barrier that tightly regulates the flow of cells, ions, and molecules from peripheral circulation into the brain. The brain receives all its iron from the peripheral circulation, with the primary pathway for iron uptake being mediated by the blood-brain barrier (BBB), composed of cerebrovascular endothelial cells (Mills et al., 2010). Almost all iron in the serum is bound to transferrin (TF), a serum iron transporter glycoprotein (Tolosano, 2015). TF-bound iron must cross the BBB to supply underlying tissues, a process facilitated by the transferrin receptor (TFRC), which is present on both the apical and basal plasma membranes of brain endothelial cells (Nielsen et al., 2023). TFRC-mediated endocytosis of iron-bound TF through clathrin-coated vesicles is primarily responsible for the transendothelial intake of iron (Mills et al., 2010). Additionally, ferritin, an iron-binding and iron-storage protein circulating in the serum, can permeate the BBB via ferritin receptors (Li et al., 2010; Fisher et al., 2007), contributing to transmembrane iron transport. Furthermore, multiple non-TF-bound iron (NTBI) transport mechanisms exist, involving divalent metal-ion transporters such as ZIP14, ZIP18, and DMT1, which are crucial for brain iron homeostasis (Knutson, 2019). Both TF-dependent and TF-independent mechanisms may contribute to iron transport across the BBB, as evidenced by hypotransferrinemic mice (with less than 1% of normal circulating transferrin) having normal amounts of brain iron (Beard et al., 2005). Once iron enters the endothelial cells, various pathways may regulate its transcytosis, involving both intracellular and abluminal iron transport (Mills et al., 2010). Moreover, different brain cell types, including neurons, astroglia, and oligodendrocytes, have specific preferences for iron transport and metabolism to maintain homeostasis. Pinpointing the defects underlying abnormal iron homeostasis in AD is a monumental task and remains poorly understood. One approach to uncovering the root cause of iron dyshomeostasis-mediated neurodegeneration in AD is to identify defects in the regulatory elements controlling receptor-mediated TF trafficking in the human brain.

Calcium, like iron, is essential for maintaining normal brain function. Calcium ions (Ca^2+^) act as crucial second messengers in cellular signaling. Disruptions in Ca^2^⁺ homeostasis and signaling are critical in the pathogenesis of neurodegenerative diseases, including AD (Alzheimer’s Association Calcium Hypothesis W, 2017). Recent studies suggest that calcium-induced iron dysregulation contributes to neurodegeneration, highlighting a complex interplay between calcium and iron signaling (Núñez and Hidalgo, 2019). In my previous research, I have identified Ca^2^⁺/Calmodulin (CAM) Dependent Protein Kinase Kinase 2 (CAMKK2) as a novel regulator of receptor-mediated transferrin (TF) trafficking across various cell types (Sabbir, 2018). CAMKK2, a serine/threonine kinase, is activated upon binding to Ca^2^⁺/CAM in response to increased intracellular Ca^2^⁺ concentrations (Kitani et al., 1997). It is expressed in a variety of cell types, including neurons (Marcelo et al., 2016) and BBB endothelial cells (Sabbir et al., 2020). Using proteomics approaches and CRISPR/Cas9-mediated CAMKK2 deletion in human embryonic kidney (HEK293) and hepatoma-derived HepG2 cells, I have demonstrated that CAMKK2 deletion results in defective TFRC-mediated endocytosis of TF, leading to abnormal accumulation of TF and altered phosphorylation of TF at multiple serine and threonine residues (Sabbir, 2018). Additionally, we found that hemizygous deletion of CAMKK2 in transformed human endothelial cells (EA.hy926) increases TF uptake and transcytosis, as well as alters subcellular trafficking of TF (Sabbir et al., 2020). Furthermore, Camkk2 knockout (Camkk2^−/−^) mice exhibited a significant increase in total TF in the brain and spinal cord, and a decrease of TF in liver tissues (Sabbir, 2020a). These findings establish a strong connection between CAMKK2 activity and iron homeostasis through regulation of receptor-mediated TF trafficking, making CAMKK2 an ideal target for study in AD brains. Based on these observations, it is hypothesized that CAMKK2, TF, and TFRC protein levels may be altered in AD brains, contributing to the dysregulated iron homeostasis observed in the diseased brains.

To address this hypothesis, the abundance of CAMKK2, TF, and TFRC proteins was analyzed in temporal cortex tissues derived from 74 AD patients and 17 age-matched CN individuals, postmortem. It is important to note that the definition of a CN individual was based on the neuropathological diagnosis reported by the respective brain repository, which may change based on subsequent neuropathological/molecular examination of the postmortem brains used in this study. Additionally, temporal cortical tissues obtained from 27 Parkinson’s disease (PD) patients were analyzed to determine if the anticipated abnormalities in CAMKK2/TF/TFRC were specific to AD or also present in other forms of dementia, specifically PD. Furthermore, a cohort of temporal/prefrontal cortical tissues derived from a total of 30 CN individuals, consisting of different age groups ranging from prenatal, postnatal, toddler, early to middle childhood, adolescents, adults, and elderly individuals, was used to assess any age-related effects on the anticipated results.

## 2 Methods

### 2.1 Postmortem human brain tissue

Frozen (−80°C) temporal cortex tissue samples (9 CN individuals, 74 AD patients, and 27 PD patients) were obtained from the University of Florida Neuromedicine Human Brain and Tissue Bank, Florida, USA. This cohort of tissue samples was previously used in another study investigating the role of Cholinergic Receptor Muscarinic 1 (CHRM1) in AD (Sabbir et al., 2022). For detailed sample information, see Supplementary Table S2 in Sabbir et al. (2022). Additionally, a cohort of prefrontal/temporal cortex tissue from 30 CN individuals of different age groups (Supplementary Table S1), ranging from prenatal, (18 weeks), postnatal (36 weeks), toddler (3 years), early to middle childhood (4–8 years), adolescents (13–14 years), adults (42 years), and elderly individuals (72–90 years) was kindly provided by Dr. Richard Deth of the Barry and Judy Silverman College of Pharmacy, Nova Southeastern University, Florida, USA. Dr. Deth obtained these samples from the National Institutes of Health (NIH) Neurobiobank (NBB) repository (Request number: 2219). Furthermore, cortical tissues from eight elderly CN individuals (mean age ± SD: 71.5 ± 12.39 years) from the NIH NBB cohort were added to the Florida Neuromedicine Human Brain CN cohort as age-matched samples for comparison with the AD and PD patient cohorts, bringing the total number of adult CN tissue samples to 17.

Hippocampus and cerebellum tissue from four CN individuals were also used to characterize brain region-specific abundance of CAMKK2 protein. The hippocampus tissue samples (deidentified sample numbers: #4456 and #1848) were provided by the NIH NBB (sample request number: 1883) and the cerebellum samples (deidentified sample numbers: #A21-006 and #A20-031) were provided by the University of Florida Neuromedicine Human Brain and Tissue Bank. Detailed information on these samples can be found in the supplementary tables published in Sabbir et al. (2022).

### 2.2 Western blotting (WB) and immunodetection

The approach used for quantifying target proteins in postmortem brain tissue by WB was previously described in detail by Sabbir et al. (2020); Sabbir et al. (2022). Increasingly, total protein staining has become the standard for normalizing Western blot quantifications, as housekeeping proteins like GAPDH may vary in disease states and thus lead to erroneous results if used for normalization (Pillai-Kastoori et al., 2020). Consequently, total cellular protein staining was employed for normalization in this study, a method rigorously validated in our previous work (Sabbir et al., 2020; Sabbir et al., 2022). Anti-CAMKK2 (catalog number: Sc-100364), anti-TF (catalog number: sc-365871), anti-TFRC (catalog number: sc-51829), anti-MAPT (catalog number: sc-58860), anti-CHRM1 (catalog number: sc-365966) and anti-GAPDH (catalog number: Sc-25778) antibodies were purchased from Santa Cruz Biotechnology. The CAMKK2, TF, and TFRC antibodies were previously used in different publications of ours and, therefore, validated (Sabbir, 2018; Sabbir et al., 2020; Sabbir, 2020a; Sabbir et al., 2022).

### 2.3 Quantification of iron content in cortical tissues

The iron content in cortical tissues was measured using a colorimetric technique with an Iron Assay Kit (Catalog Number MAK025; Sigma Inc.). Due to the unavailability of the relatively large amount of tissue (>10 mg) required for the colorimetric iron assay, the iron content was not measured in the PD cortices. In this assay, an acidic buffer was used to release iron from the tissue samples (>10 mg) by rapidly homogenizing them in 4–10 volumes of Iron Assay Buffer supplied with the kit, using a Sonic Dismembrator (Fisherbrand™ Model 120). The homogenized samples were then centrifuged at 16,000 g for 10 min at 4°C to remove insoluble material. To measure the Fe³⁺ iron, the samples were reduced using an iron reducer supplied with the kit, which allows for the determination of total iron (Fe³⁺ and Fe^2^⁺). The released iron reacts with a chromogen to produce a colorimetric product, which is measured at 593 nm and is proportional to the amount of iron present. The Fe³⁺ content was calculated as the difference between the total iron (measured in the presence of the iron reducer) and the Fe^2^⁺ content (measured with the assay buffer alone). A standard curve was generated each time the assay was performed. The total iron content of the tissue was expressed in nmol per 10 mg of tissue.

### 2.4 Statistical analysis

Statistical analysis was performed using GraphPad Prism software version 10.2.3 (403), 21 April 2024. Expression levels of the protein of interest in CN and AD/PD groups were normalized to a reference CN sample (Sample ID: A21-006; 100%) to facilitate comparison across immunoblots from independent experiments or biological replicates (Sabbir et al., 2020). Group comparisons were assessed using unpaired parametric t-tests for pairwise comparisons and one-way ANOVA (randomized) followed by Dunnett’s *post hoc* multiple comparison test for analyses involving three or more groups (Dunn, 1964; Siegel, 1956). Statistical significance was defined as *p* < 0.05. Pearson correlation tests or simple linear regression analyses were performed considering that protein expression, age at death, or postmortem intervals (PMI) or tissue iron content values approximated Gaussian distributions. The correlation coefficient (r) ranges from −1 to +1, where r = 1 indicates a perfect positive correlation, r = 0 to 1 indicates variables tend to increase or decrease together, r = 0 indicates no correlation, and r = −1 to 0 indicates one variable increases as the other decreases; r = −1 indicates a perfect negative correlation. Additionally, in simple linear regression analysis, a positive slope indicates that Y increases with increasing X, while a negative slope indicates that Y decreases with increasing X.

## 3 Results

### 3.1 Expression of CAMKK2 and TF in the hippocampus, cortex and cerebellum tissues

**FIGURE 1:**
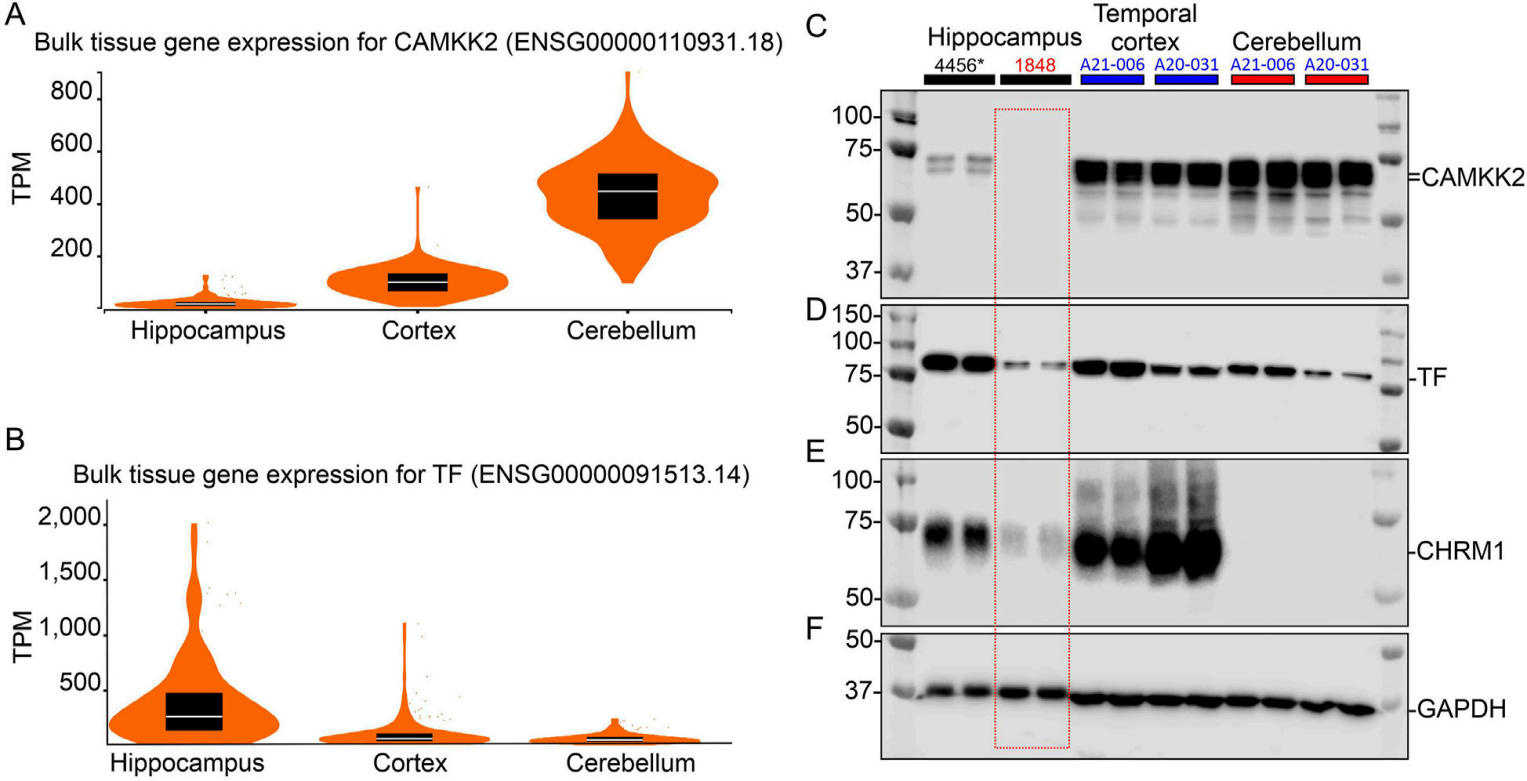
CAMKK2 mRNA and protein expression in the hippocampus, cortex, and cerebellum of human brains. **(A, B)**: Violin plots showing CAMKK2 and TF mRNA expression in primary human brain tissues. TPM: Transcripts per million. The data used for this analysis were obtained from the GTEx portal on 9 June 2024. Sample sizes for CAMKK2 and TF are as follows: hippocampus N = 197, cortex N = 255, cerebellum N = 251/241 respectively. Box plots are shown as median, 25th, and 75th percentiles. For representative images of coronal sections of the human brain showing the sample collection regions from respective brain areas, please see Sabbir et al. (2022). **(C–F)**: Immunoblots showing the abundance of CAMKK2 **(C)**, TF **(D)**, CHRM1 **(E)**, and GAPDH **(F)** in the hippocampus, temporal cortex, and cerebellum samples derived from different CN individuals. The human subject identification numbers are written on the top panel. The asterisk indicates a neonatal brain whose sex was not reported. Blue and red highlights indicate female and male sex, respectively. The red-dotted rectangle indicates abnormal levels of CAMKK2, TF, and CHRM1 proteins in the hippocampus tissue of CN sample #1848 compared to sample #4456.

CAMKK2 and TF proteins have been reported to be expressed in various regions of the mammalian brain, involving different brain cell types (Sabbir, 2018; Abe et al., 2022; Sabbir, 2020b). The gene expression data archived in the human Genotype-Tissue Expression (GTEx) database reveals differential expression patterns of *CAMKK2* and *TF* across brain regions, with transcripts per million (TPM) levels ranked as cerebellum > cortex > hippocampus for *CAMKK2* (Figure 1A) and hippocampus > cortex > cerebellum for *TF* (Figure 1B). Based on these information, immunoblotting was performed to assess the abundance of *CAMKK2* and *TF* proteins in the hippocampus, cortex, and cerebellum tissues obtained postmortem from cognitively normal (CN) individuals (Figures 1C–F). Immunoblotting showed the presence of *CAMKK2* as immunoreactive bands of approximately 70–75 kDa (kDa) in all three regions (Figure 1C), corresponding to the known *CAMKK2* isoforms, as validated in previous studies (Sabbir, 2020b). Similarly, *TF* was detected as an ∼80 kDa protein in the hippocampus, cortex, and cerebellum (Figure 1D), consistent with earlier findings in postmortem human tissues (Sabbir, 2018). Notably, *CAMKK2*-specific bands were absent in sample #1848, which also showed reduced levels of both *TF* and the muscarinic cholinergic receptor type 1 (*CHRM1*) (Figures 1C–F, red dotted rectangle). Though, sample #1848 was classified as CN by the NIH NBB, the reduced expression of *CHRM1* in the hippocampus relative to sample #4456 suggests the possibility of an undiagnosed neurodegenerative condition, as *CHRM1* loss in the hippocampus has been linked to such conditions (Sabbir et al., 2022).

### 3.2 CAMKK2 and TF protein levels remain unaffected by aging, while TFRC levels decline in older individuals

The abundance of CAMKK2 and TF proteins were not significantly affected by the aging process, while TFRC protein levels tended to decline in older individuals. A cohort of prefrontal cortex tissues (Table 1, N = 30), primarily from Brodmann Area 10 (BA10), derived from CN individuals with ages ranging from prenatal (18 weeks) to elderly (72–90 years) was analyzed.

**TABLE 1:** Summary of the major findings observed in terms of statistical comparison of CAMKK2, TF, and TFRC protein levels, iron content, age, gender, and PMI factors in CN, AD, and PD.

Protein/Factor	Young vesus Old	CN	AD	PD
CAMKK2	Unaltered with increasing age	Normal	Significantly decreased	Significantly decreased
TF	Unaltered with increasing age	Normal	Significantly decreased	Significantly decreased
TFRC	Significantly decreased with increasing age	Normal	Significantly decreased	Significantly decreased
Iron	Not done	Normal	Significantly increased	Not done

Protein/Factor	Age	Postmortem Interval (PMI)	Tissue Iron content
CAMKK2	CN: not significant/AD: not significant/PD: not significant	CN: not signifcant/AD : significant/PD: not significant	CN: not signifcant/ AD: not significant
TF	CN: not significant/AD: significant/PD: significant	CN: not signifcant/AD : not sifgnificant/PD: not significant	CN: not signifcant/ AD: significant
TFRC	CN: not significant/AD : not significant/PD: not significant	CN: not signifcant/AD : significant/PD: not significant	CN: not signifcant/ AD: significant
Age			CN: not signifcant/ AD: significant

**FIGURE 2:**
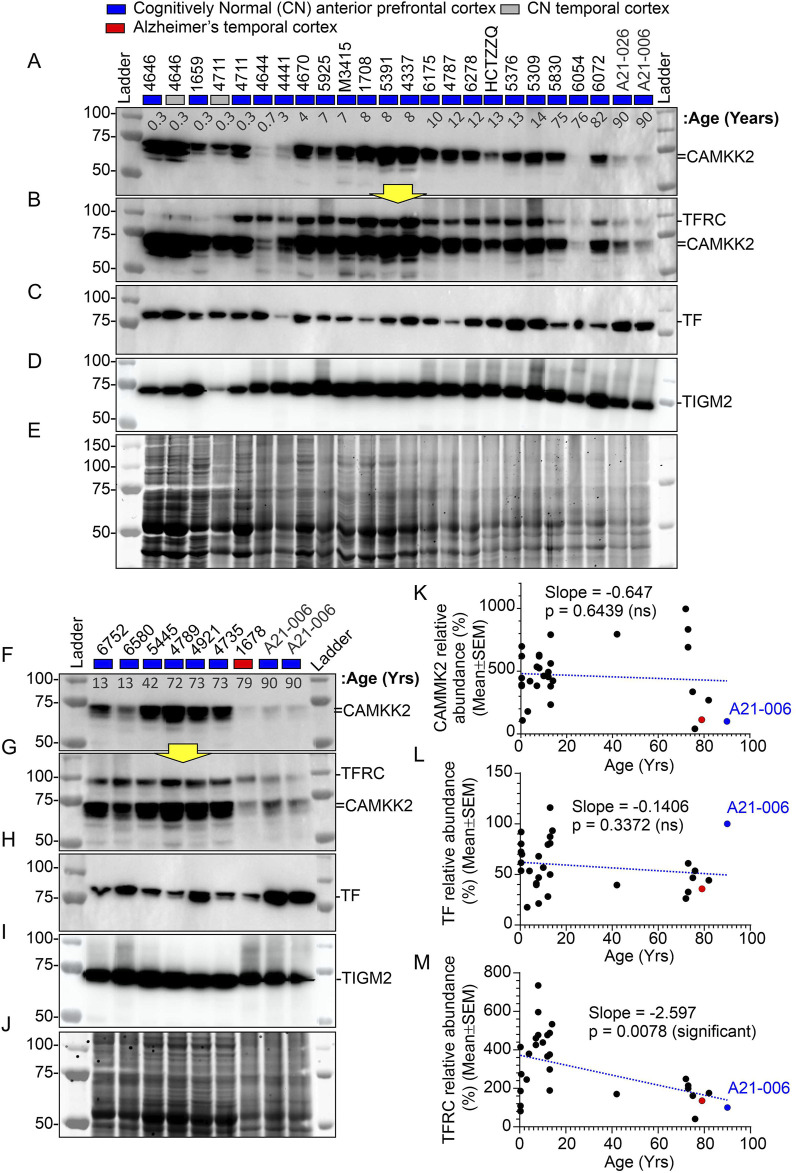
Abundance of CAMKK2, TF, and TFRC proteins in the anterior prefrontal/temporal cortex of CN individuals of different ages, from infants to adolescents, adults, and the elderly. **(A–J)**: Immunoblots showing the abundance of CAMKK2 **(A, F)**, TFRC **(B, G)**, TF **(C, H)**, and transglutaminase 2 (TGM2) **(D, I)** in individuals of different age groups. Panels E and J represent Oriole-stained SDS-PAGE gels showing the protein loading corresponding to the respective top panel immunoblots. Yellow arrows indicate that the corresponding blot was incubated with the TFRC antibody without stripping the previously applied CAMKK2 antibody. The age at death of the individuals corresponding to the brain samples is shown in years in the top panel below sample numbers. The CN sample #A21-006 was loaded twice and used in each SDS-PAGE as a reference sample for normalization of the blots. The relative amount of respective proteins in each sample was calculated as a percentage of the CN control sample #A21-006 used to normalize all samples in the same blot. This approach ensures that different immunoblots generated under different experimental conditions during the study are comparable. **(K–M)**: Scatter plots showing the relative abundance (average of three independent experiments with one replicate each) of CAMKK2, TF, and TFRC proteins in individuals of different age groups. The red and blue filled data points indicate AD patient sample and the CN reference sample (#A21-006), respectively. The blue dotted lines in K-M scatter plots represent the simple linear regression line of the data, and the *p*-value represents whether the slope is significantly non-zero. If the slope is significantly non-zero (*p*-value <0.05), it suggests that the independent variable (e.g., age) has a significant effect on the dependent variable (e.g., protein abundance). ns: not significant. The regression lines were drawn using GraphPad Prism software.

The abundance of CAMKK2, TF, and TFRC proteins in the cortical regions of the human brain postmortem was quantified using immunoblotting. The quantification was based on immunoblots (Figures 2A–J), followed by plotting the mean CAMKK2, TF, and TFRC protein levels with respect to age (Figures 2K–M, respectively). Simple linear regression analysis revealed no significant difference in CAMKK2 and TF protein levels with increasing age (CAMKK2 vs. age: Slope = −0.6477, *p*-value = 0.6439, R-squared = 0.0077; TF vs. age: Slope = −0.1406, *p*-value = 0.3372, R-squared = 0.0329). However, TFRC protein levels in the temporal cortex significantly decreased with age (TFRC vs. age: Slope = −2.597, *p*-value = 0.0078, R-squared = 0.2271), indicating that the reduction in TFRC protein levels may depend on increasing age (Figure 2M). Furthermore, a comparison of CAMKK2, TF, and TFRC protein levels with postmortem interval (PMI) using simple linear regression (CAMKK2 vs. PMI: Slope = −3.373, *p*-value = 0.3895, R-squared = 0.02656; TF vs. PMI: Slope = 0.1102, *p*-value = 0.7906, R-squared = 0.002561; TFRC vs. PMI: Slope = 0.3863, *p*-value = 0.8948, R-squared = 0.00063) revealed that the alterations in protein abundance within the temporal cortex tissues of old and young CN individuals were not dependent on PMIs (Table 1).

Overall, these findings indicate that CAMKK2 and TF protein levels remain relatively unchanged with increasing age, while TFRC protein levels decrease (Table 1). These results encourage further study of these protein levels in Alzheimer’s brains to better understand the disease pathology.

### 3.3 Characterization of AD and PD brain tissue cohorts using microtubule-associated protein tau (MAPT) profiling

**FIGURE 3:**
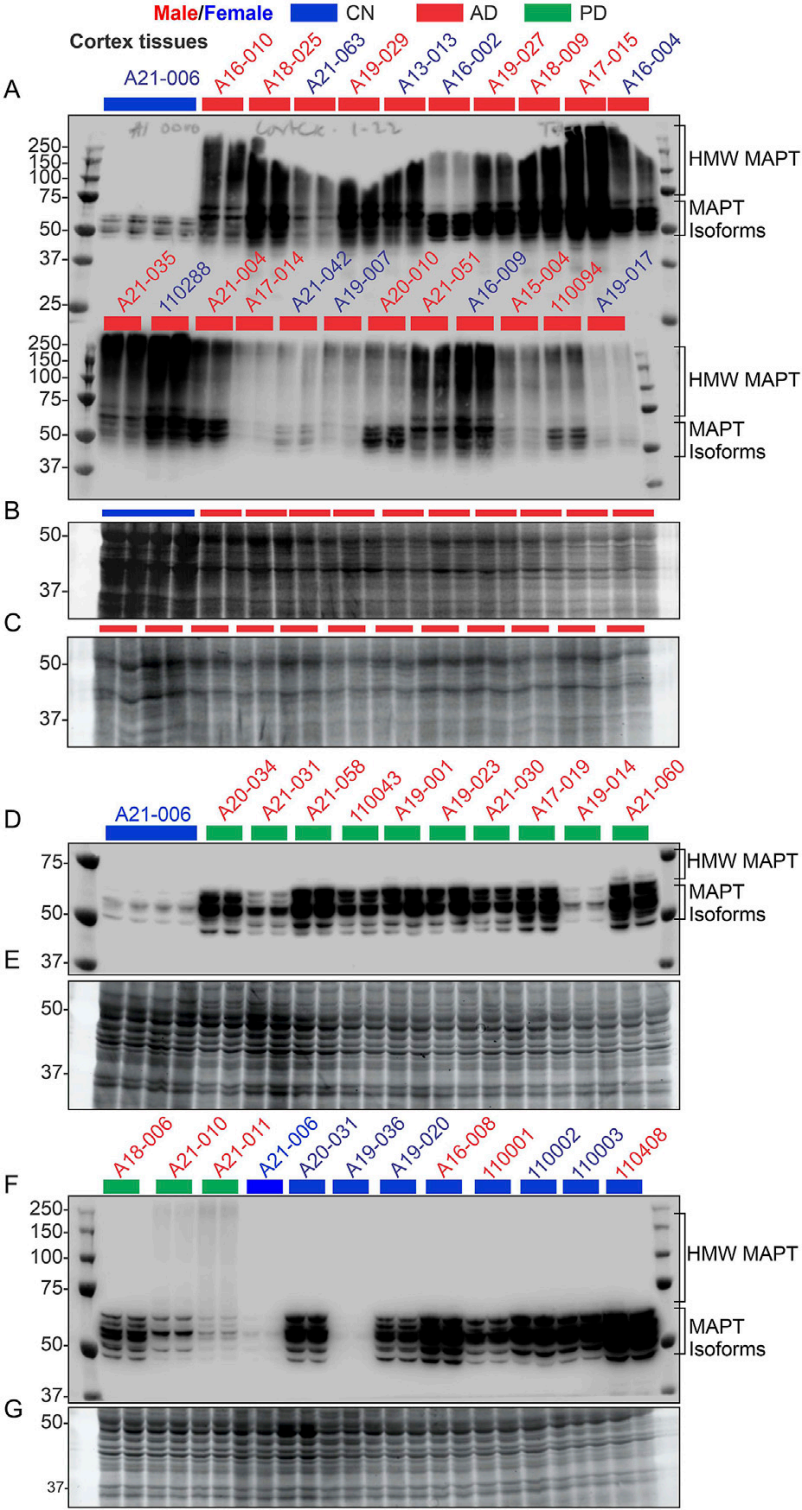
Verification of the nature of the disease in AD patient samples using the MAPT pathological marker, compared with CN and PD samples. **(A, D, F)**: Immunoblots showing the relative abundance of MAPT in temporal cortex tissues derived from postmortem human brains. The sample identification numbers are provided on the top panel of each immunoblot. Males and females are highlighted in blue and red, respectively. CN, AD, and PD samples are color-coded blue, red, and green, respectively. Panels **(B, C)** and **(E, G)** represent Oriole-stained SDS-PAGE gels showing the protein loading corresponding to the respective top panel immunoblots. HMW: high molecular weight.

A cohort of 17 CN, 74 AD, and 27 PD cortical tissue samples, derived from postmortem brains [Supplementary Table S2 in Sabbir et al. (2022)], were used to analyze CAMKK2, TF, and TFRC protein abundance and iron contents. The primary characterization of these tissue samples as CN, AD, or PD was performed by the respective brain repositories [Supplementary Table S2 in Sabbir et al. (2022)]. However, an immunoblotting using an anti-MAPT antibody was conducted (Figure 3) to further validate the disease nature of these tissues, specifically AD, based on the hypothesis that MAPT aggregates (polymerization), commonly known as neurofibrillary tangles (NFTs), a hallmark pathological feature of AD brains (Binder et al., 2005), would be expected in the AD cortical tissues but not in the PD and CN tissues.

Six MAPT isoforms are present in the human brain and their molecular weight ranges between 45 and 65 kDa (Park et al., 2016). MAPT aggregates appear as a high molecular weight smear in Western blot (Watanabe et al., 1999; Sergeant et al., 2001; Wu et al., 2021; Zhou et al., 2018; Tarutani et al., 2021; Li et al., 2021; Santa-Maria et al., 2012; Chukwu et al., 2018; Humpel, 2015; Jackson et al., 2016; Berger et al., 2007; Takaichi et al., 2021). Immunoblotting revealed the appearance of multiple bands of varying intensities in the 45–65 kDa regions in the immunoblots derived from the CN (n = 9) and PD (n = 13) tissues (Figures 3A–G), indicating the presence of MAPT isoforms. In contrast, MAPT appeared as a high molecular weight smear ranging from 50 to 250 kDa in the 22 AD samples that were analyzed (Figures 3A–C). The presence of the high molecular weight MAPT smear in the AD samples indicates NFT pathology, which was not present in the CN and PD samples. This further confirmed the disease nature of the AD tissue samples used in this study. It is important to note that some of the PD patients also exhibited a faint high molecular weight MAPT signal in the immunoblots (Sample IDs A21–010 and A21-011), indicating the presence of NFT pathologies. Overall, these findings confirmed the presence of distinct AD pathologies in the AD patient cohorts.

### 3.4 Significant reduction of CAMKK2 in AD patient temporal cortices compared to age-matched CN individuals

**FIGURE 4:**
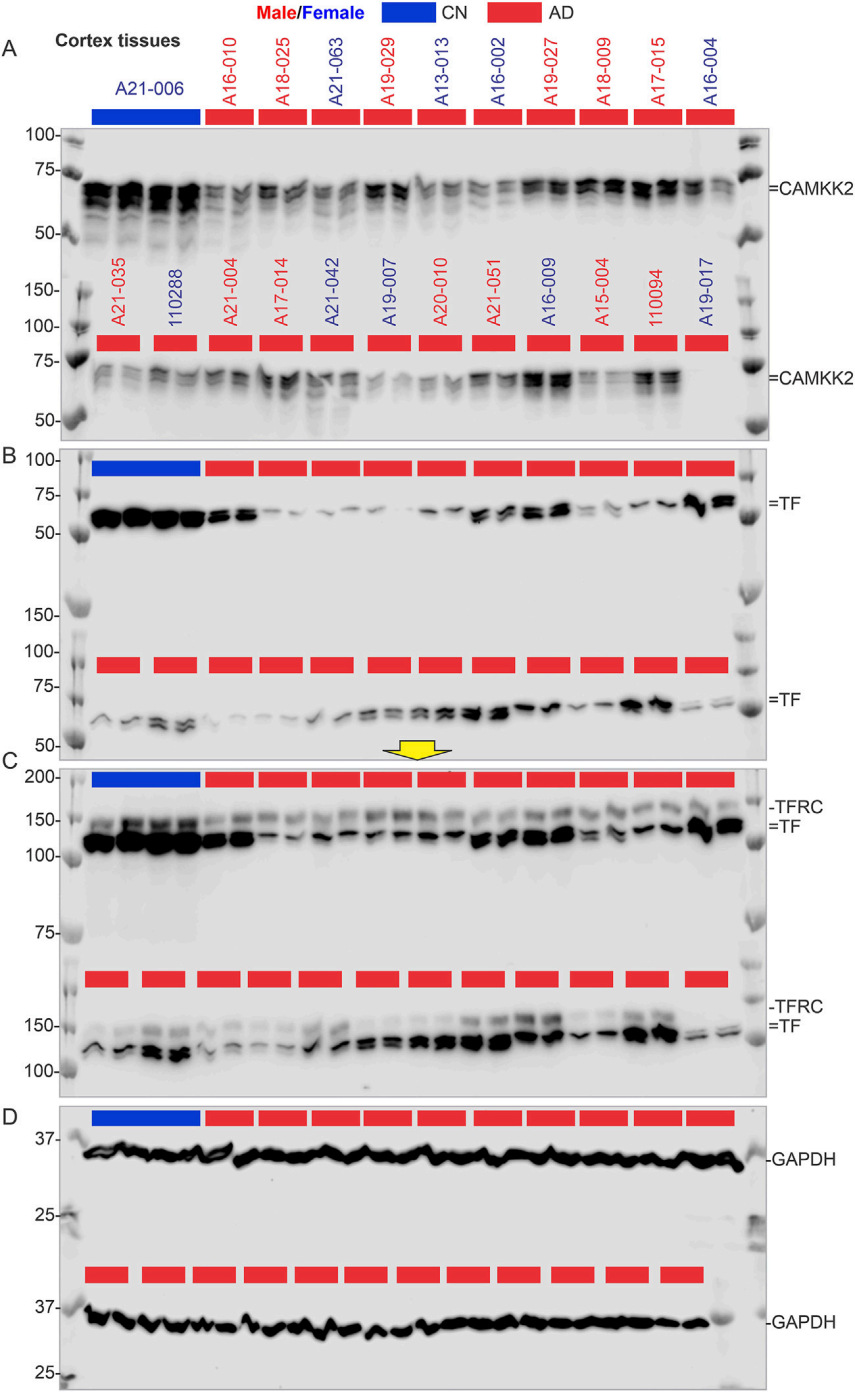
CAMKK2, TF, TFRC, and GAPDH protein levels in the temporal cortex of AD patients, with a CN individual (A21-006) used as a reference sample for normalization. **(A–D)**: Immunoblots showing the relative abundance of CAMKK2 **(A)**, TF **(B)**, TFRC **(C)**, and GAPDH **(D)** in temporal cortex tissues derived from human brains, postmortem. The sample identification numbers are provided on the top panel of each immunoblot. Males and females are highlighted in blue and red, respectively. CN and AD samples are color-coded blue and red, respectively. The yellow arrow indicates that the corresponding immunoblot was incubated with a different primary antibody (TFRC) without stripping the previously used primary antibody (TF). Protein loading for samples in immunoblots A-D is verified in the corresponding Oriole-stained gel shown in Figure 3B.

Immunoblotting was performed to quantify CAMKK2 protein levels in the temporal cortex tissues derived from AD patients and CN individuals, postmortem (Figures 4A, D, and Supplementary Figures S1A, D). Quantification based on the immunoblots revealed a significant reduction (*p* < 0.0001) of CAMKK2 protein levels in the AD cortices compared to CN individuals (Figure 6A). The abundance of cortical CAMKK2 protein in male (n = 36) and female (n = 38) AD patients exhibited no significant difference (*p* = 0.2505) in an unpaired *t*-test (Figure 6B), indicating this phenomenon is not affected by gender. When CAMKK2 protein levels in AD and CN patients were plotted based on their age at the time of death, simple linear regression analysis indicated that the variation in cortical abundance of CAMKK2 protein is not significantly associated with age in both CN (CAMKK2 in CN vs. Age: Slope: -9.245, *p*-value = 0.0929, R-squared = 0.01768) and AD (CAMKK2 in AD vs. Age: Slope: 0.01904, *p*-value = 0.9601, R-squared = 0.00003497) patients (Figures 6C, D). Interestingly, the plot involving CAMKK2 protein levels versus age in AD patients revealed a cluster of individuals with very low CAMKK2 protein levels in the age range between 70 and 80 years. This prompted segmenting the data into 10-year intervals, revealing a significant difference between CAMKK2 protein levels in those who died at the age of 70–80 compared to <70, <90, and <100 age groups (Figure 6E). Additionally, CAMKK2 protein levels in AD and CN patients were also plotted based on the PMI. Simple linear regression analysis indicated that the variation in CN individuals’ temporal cortical CAMKK2 protein levels was not significantly related to their PMI (CAMKK2 in CN vs. PMI: Slope: 6.323, *p*-value = 0.9129, R-squared = 0.0008250; Figure 6F). In contrast, variation in the CAMKK2 protein levels in AD cortices was significantly related to their PMI (CAMKK2 in AD vs. PMI: Slope: 6.323, *p*-value = 0.9129, R-squared = 0.0008250; Figure 6G).

Overall, these results indicate a significant reduction in CAMKK2 protein levels in AD cortices compared to age-matched CN individuals, which may be associated with an age-related phenomenon. However, the possibility that reduced CAMKK2 in AD brains is due to PMI can be considered a false result since this was not observed in CN brains and PD brains (Table 1), as described in subsequent sections. Additionally, the distribution of PMI values was not significantly different and was comparable; for example, the mean ± standard deviation for AD (N = 74), PD (N = 27), and CN (N = 17) were 11.76 ± 7.35, 10 ± 5.6, and 15.24 ± 12.12, respectively.

### 3.5 Significant reduction of CAMKK2 in PD patient temporal cortices compared to age-matched CN individuals

**FIGURE 5:**
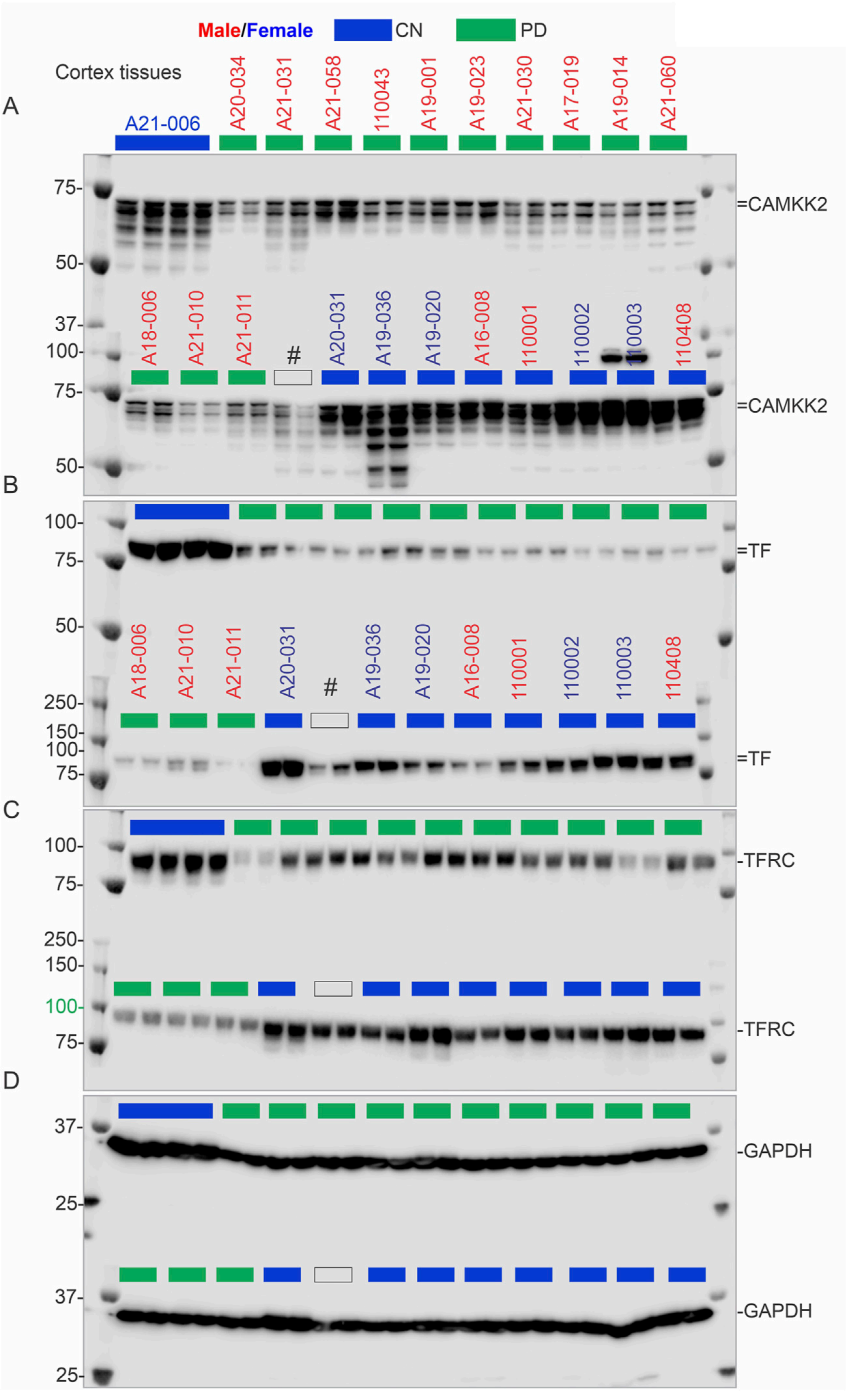
CAMKK2, TF, TFRC, and GAPDH protein levels in the temporal cortex of CN individuals, including the reference CN individual (A21-006), and PD patients. **(A–D)**: Immunoblots showing the relative abundance of CAMKK2 **(A)**, TF **(B)**, TFRC **(C)**, and GAPDH **(D)** in temporal cortex tissues derived from human brains, postmortem. The sample identification numbers are provided on the top panel of each immunoblot. Males and females are highlighted in blue and red, respectively. CN, AD, and PD samples are color-coded blue, red and green, respectively. Protein loading for samples in immunoblots A-D is verified in the corresponding Oriole-stained gel shown in Figure 3E.

**FIGURE 6:**
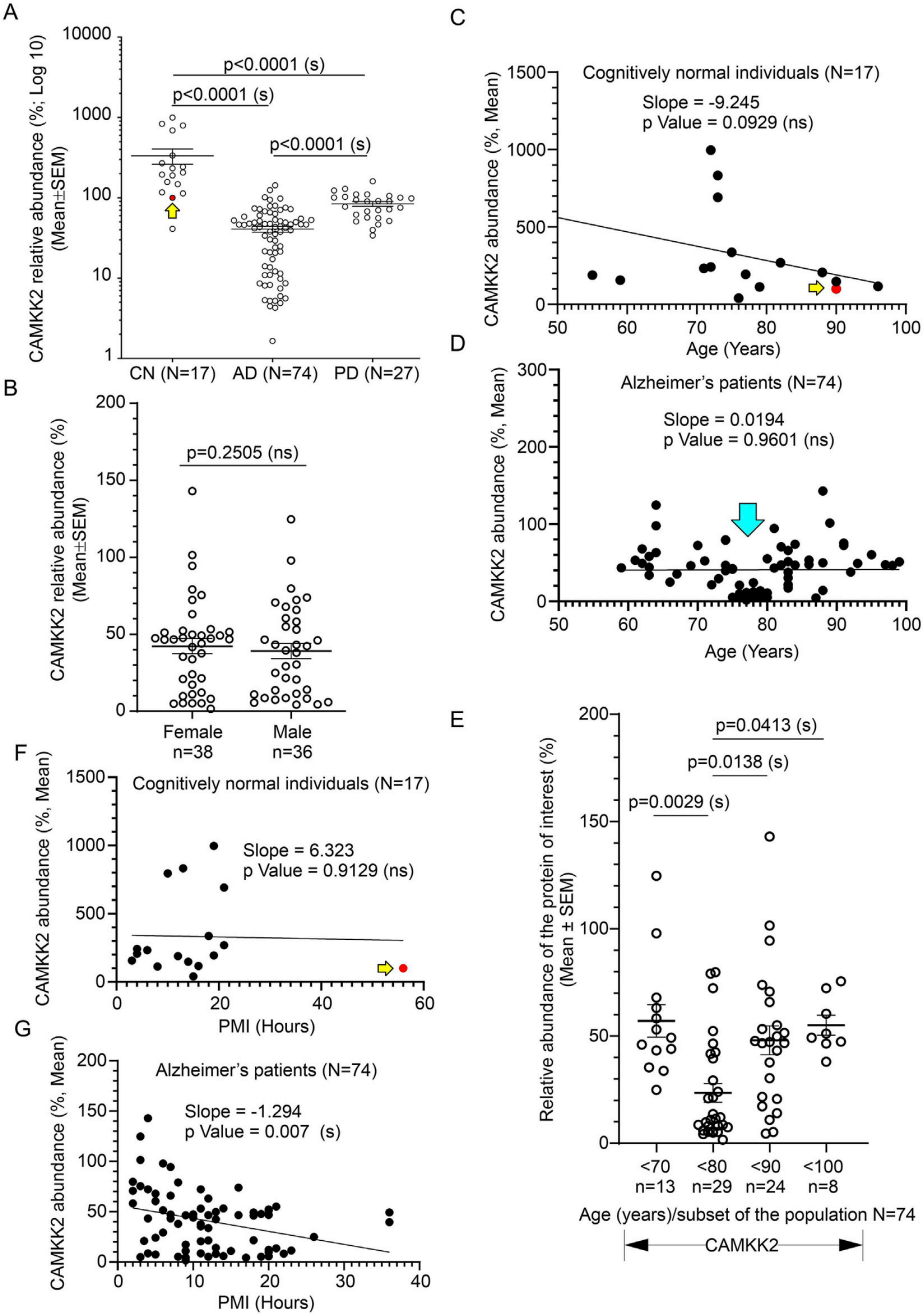
Alteration of CAMKK2 in the temporal cortex of AD and PD patients compared to CN individuals, postmortem, and correlation with age of death and PMI. **(A)**: Scatter plot showing CAMKK2 protein levels in the temporal cortex tissues obtained from 17 CN individuals and 74 AD and 27 PD patients, postmortem. The mean protein abundance value (%) compared to a reference CN sample (#A21-006) was obtained by averaging the protein abundance values from 3 independent experiments (immunoblotting) with 2 replicates in each, using the same tissue lysates. The red filled data point and the yellow arrow in the scatter plots **(A, C, F)** indicate the data point referring to the reference protein sample (#A21-006). Data are presented as Mean ± SEM. *p*-values by Ordinary One-Way ANOVA followed by Dunnett’s Multiple Comparisons test. **(B)**: Scatter plot showing temporal cortical CAMKK2 abundance in male (n = 36) and female (n = 38) patients within the AD cohort (N = 74). *p*-value by unpaired *t*-test. Ns = not significant. **(C, D, F, G)**: Scatter plots showing the abundance of CAMKK2 proteins (%, Mean from **(A)** in the temporal cortex combined with the age variables and PMI of the CN individuals (C and F, respectively) and AD patients (D and G, respectively). The cyan arrow in D indicates a cluster of data showing significantly low (<50% compared to the CN reference sample) CAMKK2 protein levels in patients who died within 70–80 years of age. **(E)**: Scatter plot showing the CAMKK2 protein levels (% mean from **(A)** in the temporal cortex derived from different age groups of AD patients. *p*-value by One-Way ANOVA followed by Dunnett’s Multiple Comparisons test. The regression lines were drawn using GraphPad Prism software.

Immunoblotting was performed to quantify CAMKK2 protein levels in the temporal cortex tissues derived from PD (N = 27, 5 female and 22 male) patients postmortem (Figures 5A, D). Quantification based on the immunoblots revealed a significant reduction (*p* < 0.0001) of CAMKK2 protein levels in the PD cortices compared to CN individuals (Figure 6A). Although the male-to-female ratio of the PD patients was disproportionate and not ideal for statistical comparison, an unpaired *t*-test with Welch’s correction revealed no significant difference (*p* = 0.2763) between the mean CAMKK2 levels in male and female PD patients’ cortices. Furthermore, simple linear regression analysis indicated that the variations in PD patients’ temporal cortical CAMKK2 protein levels were not significantly related to their age (CAMKK2 in PD vs. age: Slope: 0.1032, *p*-value = 0.9025, R-squared = 0.0006124; Supplementary Figure S2A) or PMI (CAMKK2 in PD vs. PMI: Slope: -1.403, *p*-value = 0.1779, R-squared = 0.07137; Supplementary Figure S2B), indicating a disease-specific event not depending on PMI or age variables.

Overall, these findings indicate that CAMKK2 protein levels in the PD temporal cortices are significantly reduced (Table 1). However, this reduction was not related to age or PMI, an observation similar to what was found in the cortices of AD patients.

### 3.6 Significant reduction of TF and TFRC in AD patients’ temporal cortices compared to age-matched CN individuals

**FIGURE 7:**
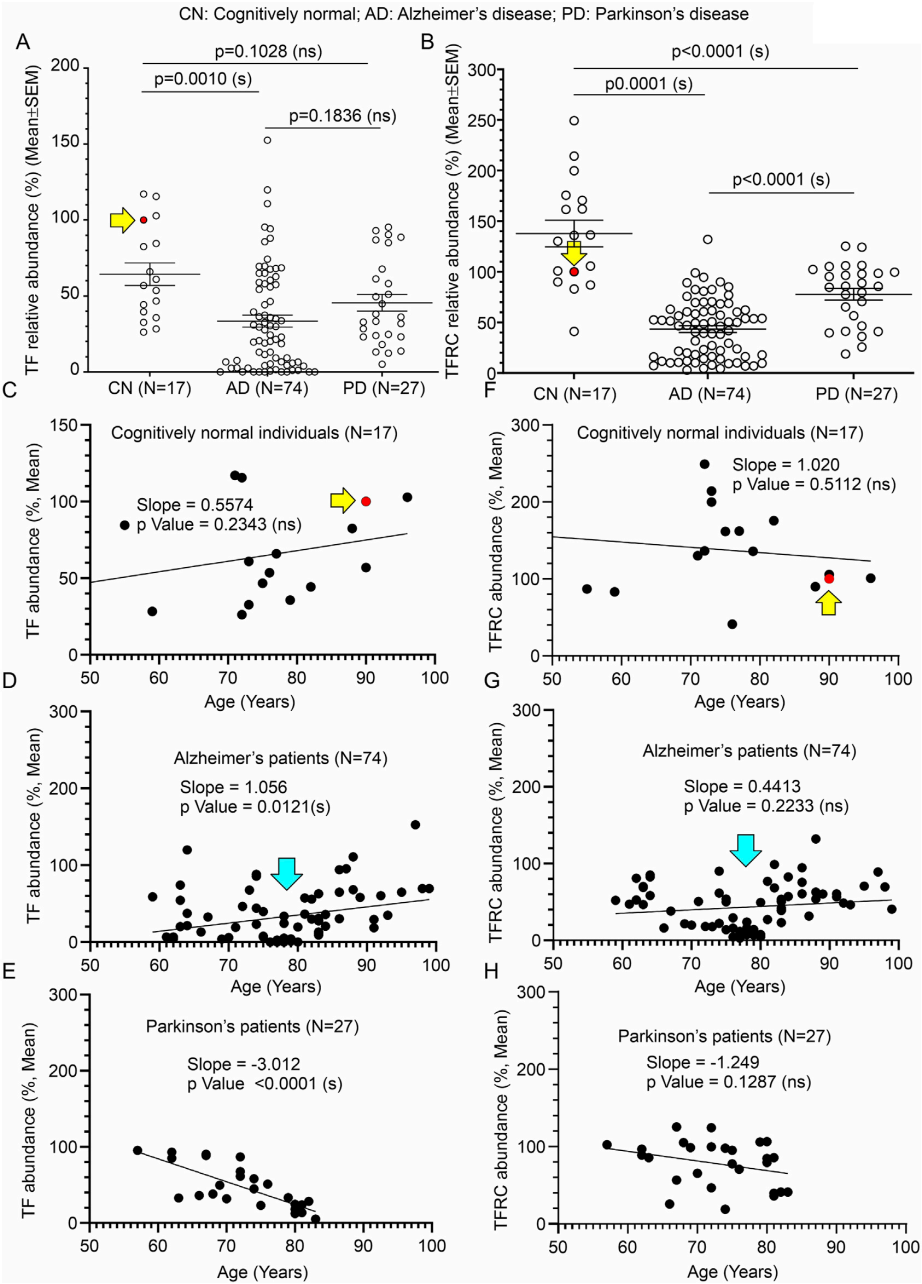
Alteration of TF and TFRC in the temporal cortex of AD and PD patients compared to CN individuals, postmortem, and correlation with age of death. **(A, B)**: Scatter plots showing TF and TFRC protein levels in the temporal cortex tissues obtained from 17 CN individuals, 74 AD patients, and 27 PD patients, postmortem. The mean protein abundance value (%) compared to a reference CN sample (#A21-006, solid red data point indicated by a yellow arrow) was obtained by averaging the protein abundance values from 3 independent experiments (immunoblotting) with 2 replicates each, using the same tissue lysates. The solid red filled data point and the yellow arrow in the scatter plots **(A, B, C, F)** indicate the data point referring to the reference protein sample (#A21-006). Data are presented as Mean ± SEM. *p*-values were determined by Ordinary One-Way ANOVA followed by Dunnett’s Multiple Comparisons test. **(C–H)**: Scatter plots showing the abundance of TF **(C–E)** and TFRC **(F–G)** proteins (%, Mean from **(A)** in the temporal cortex combined with the age variables of the CN individuals **(C, F)**, respectively, AD patients **(D, G)**, respectively, and PD patients **(E, H)**, respectively). The cyan arrows in D and G indicate a cluster of data showing significantly low (<50% compared to the CN reference sample) CAMKK2 protein levels in AD patients who died within 70–80 years of age. The regression lines were drawn using GraphPad Prism software.

Immunoblotting was performed to quantify TF and TFRC protein levels in the temporal cortex tissues derived from AD (Figures 4B, C, and Supplementary Figures S1B, C respectively) and PD (5B&C respectively) patients, postmortem. Quantification based on the immunoblots revealed a significant reduction of both TF and TFRC protein levels in the AD cortices (*p* = 0.0010 and <0.0001 respectively) but only reduction of TFRC in PD (*p* < 0.001) patients compared to CN individuals (Figures 7A, B). The cortical TF and TFRC contents in male (n = 36) and female (n = 38) AD patients exhibited no significant difference (*p* = 0.5720 and 0.2505 respectively) in an unpaired *t*-test (Supplementary Figures S2C, D), indicating this phenomenon is not affected by gender.

When TF protein levels in AD, PD, and CN patients were plotted based on their age at the time of death, simple linear regression analysis indicated that the variation in cortical abundance of TF protein is not significantly associated with age in CN (TF in CN vs. Age: Slope: 0.5574, *p*-value = 0.2343, R-squared = 0.09289) individuals but is significantly associated with age in AD (TF in AD vs. Age: Slope: 1.056, *p*-value = 0.0121, R-squared = 0.08428) and PD (TF in PD vs. Age: Slope: -3.012, *p*-value <0.0001, R-squared = 0.5757) patients (Figures 7C–E). Interestingly, though in AD and PD patients decrease in TF content significantly correlated with age, the slopes indicate an increase in TF content with age in AD patients, while in PD patients it is the reverse (Figures 7D, E). Plotting TFRC content with age followed by simple regression analysis revealed that these variations are not significantly interdependent in CN individuals (TFRC in CN vs. Age: Slope: 1.020, *p*-value = 0.5112, R-squared = 0.02930), AD (TFRC in AD vs. Age: Slope: 0.4413, *p*-value = 0.2233, R-squared = 0.02052), and PD (TFRC in PD vs. Age: Slope: -1.249, *p*-value = 0.1287, R-squared = 0.08986) patient groups (Figures 7F–H). Furthermore, the plots involving TF/TFRC protein levels versus age in AD and PD patients revealed a cluster of individuals with very low TF/TFRC protein levels in the age range between 70 and 80 years. Segmentation of this data into 10-year intervals revealed a significant difference between TF/TFRC protein levels in those who died at the age of 70–80 compared to <70, <90, and <100 age groups (Supplementary Figure S2E). Overall, these findings indicate that both TF and TFRC in the cortex of AD and PD patients significantly decreased, which could be due to disease modification. Furthermore, based on the trend line in the regression analysis (Figures 7C–H) and age-wise segmentation of the datasets (Supplementary Figures S2E), it may be concluded that AD patients with high TF content lived longer compared to those with low cortical TF content. The TFRC abundance also showed a somewhat similar trend.

Plotting TF and TFRC protein levels in CN, AD, and PD individuals with PMI followed by simple linear regression analysis (Supplementary Figures S3A–F) indicated that the variation in cortical TF with PMI is not significantly associated in CN individuals (TF in CN vs. PMI: Slope: 0.1971, *p*-value = 0.7659, R-squared = 0.006094; Supplementary Figure S3A), AD (TF in AD vs. PMI: Slope: -0.7688, *p*-value = 0.1545, R-squared = 0.02796; Supplementary Figure S3B) and PD (TF in PD vs. PMI: Slope: -0.0725, *p*-value = 0.9248, R-squared = 0.0003639; Supplementary Figure S3C) patients. In contrast, TFRC protein level was found to be significantly associated with PMI in AD (TFRC in AD vs. PMI: Slope: -1.074, *p*-value = 0.0174, R-squared = 0.07609; Supplementary Figure S3E) but not in the CN (TFRC in CN vs. PMI: Slope: 0.3298, *p*-value = 0.7782, R-squared = 0.005451; Supplementary Figure S3D) and PD (TFRC in PD vs. PMI: Slope: -0.9503, *p*-value = 0.3753, R-squared = 0.03156; Supplementary Figure S3F) patient groups.

In summary, TF and TFRC protein levels in the temporal cortices of AD patients were significantly reduced compared to age-matched CN individuals (Table 1). This reduction was not gender-dependent. Age-related analyses showed a significant increase in TF levels with age in AD patients and a decrease in PD patients, while TFRC levels did not show significant age-related trends in any group (Table 1). Notably, low TF/TFRC levels were observed in AD and PD patients within the 70-80 age range, indicating a potential critical period for disease progression. PMI significantly impacted TFRC levels in AD patients but not TF levels, suggesting the complexity of postmortem changes or the possibility that this is an artifact since the PMI values were not significantly different among the three groups. If PMI affected the integrity of one protein in one group, it would be expected to impact the others as well.

### 3.7 Significantly increased iron content in the AD brain compared to age-matched CN individuals

**FIGURE 8:**
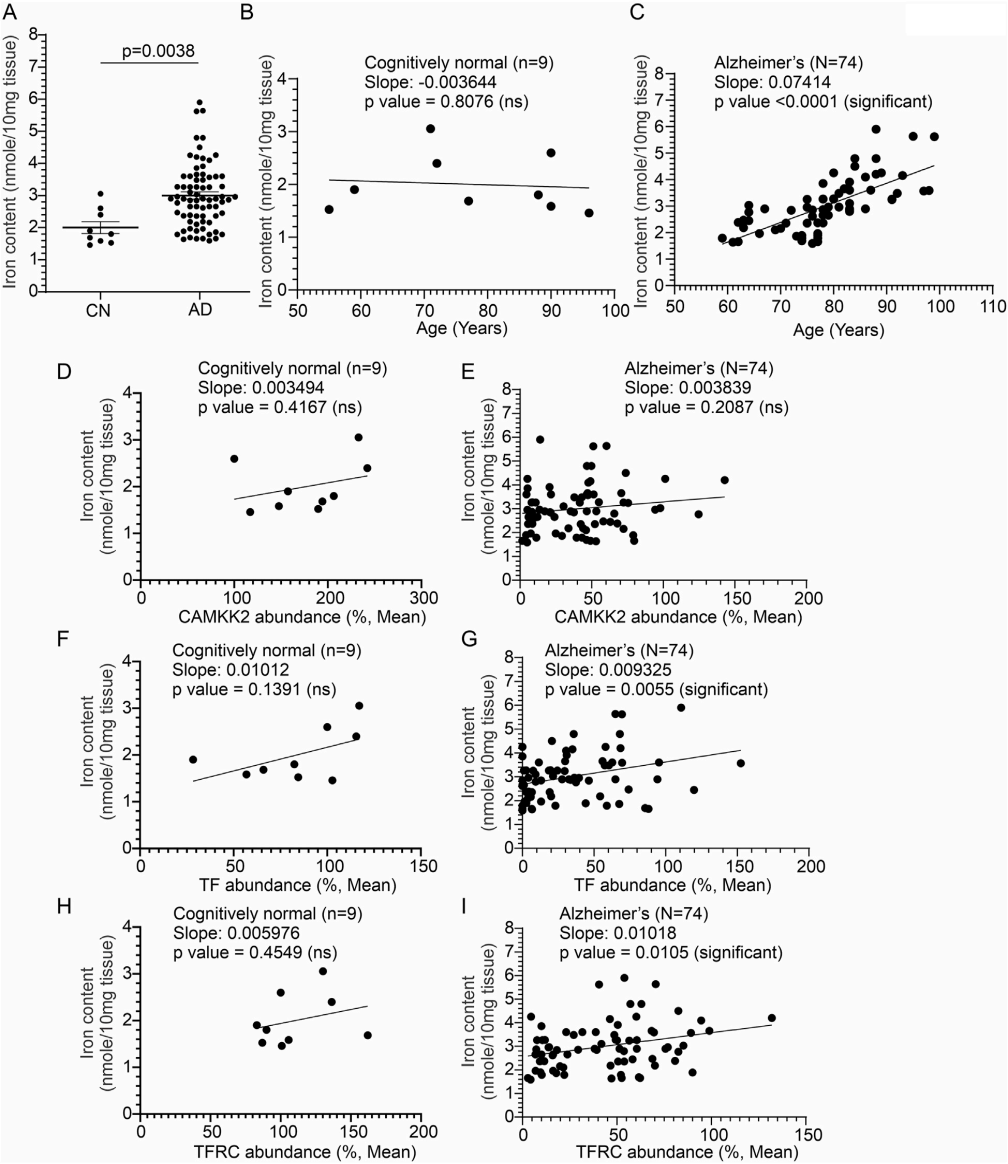
Altered temporal cortical tissue iron content in AD patients compared to CN individuals, postmortem, correlated with altered TF and TFRC protein content and the age of death. **(A, B)**: Scatter plots showing total iron content in the temporal cortex tissues obtained from 17 CN individuals and 74 AD patients, postmortem. *p*-values were determined by unpaired *t*-test. **(B, C)**: Scatter plots showing the temporal cortex tissue iron content combined with the age variables of the CN individuals **(B)** and AD patients **(C)**. **(D–I)**: Scatter plots showing the temporal cortex tissue iron content combined with the cortical CAMKK2, TF, and TFRC protein levels of the CN individuals and AD patients. The regression lines were drawn using GraphPad Prism software.

The iron content in CN (n = 9) and AD patients’ (N = 74) cortices was measured by colorimetric analysis to correlate with altered CAMKK2, TF, and TFRC protein levels. The iron content in the temporal cortex tissue was found to be significantly increased (*p*-value = 0.0038, Figure 8A) compared to age-matched CN individuals. No gender-specific difference was found in cortical iron abundance within the AD patient cohorts. Plotting iron content and age in CN (n = 9) and AD patients (n = 74) revealed a significant positive correlation between age and iron content in the AD brains (Iron in AD brains vs. Age: Slope = 0.07414, *p*-value <0.0001, R-squared = 0.4889; Figure 8C), but not in the CN brains (Iron in CN brains vs. Age: Slope = −0.003644, *p*-value = 0.8706, R-squared = 0.009060; Figure 8B). Furthermore, plotting CAMKK2, TF, and TFRC contents with iron content in the temporal cortices revealed no significant correlation between these factors and iron content in CN brains (Iron in CN brains vs. CAMKK2/TF/TFRC: Slope = 0.003494/0.01012/0.005976, *p*-value = 0.4167/0.1391/0.4549, R-squared = 0.09618/0.2846/0.08205 respectively; Figures 8D, F, H). In contrast, in the AD brains, TF and TFRC contents were found to be positively correlated with iron content (Iron in AD brains vs. TF/TFRC: Slope = 0.009325/0.01018, *p*-value = 0.0055/0.0105, R-squared = 0.1024/0.08746 respectively; Figures 8G, I), whereas CAMKK2 (Iron in AD brains vs. CAMKK2: Slope = 0.004870, *p*-value = 0.2087, R-squared = 0.02186; Figure 8E) was not found to be significantly correlated with iron content.

In conclusion, the findings reveal a significant increase in iron content in the temporal cortices of AD patients compared to CN individuals, with age being a significant factor in the AD group (Table 1). Additionally, TF and TFRC levels show positive correlations with iron content in AD brains, suggesting their potential role in iron dysregulation in Alzheimer’s disease.

## 4 Discussion

Iron dysregulation is a well-established feature of AD pathology (Liu et al., 2018; Levi et al., 2024; Spotorno et al., 2020), and this study provides the first evidence linking abnormally low levels of CAMKK2, TF, and TFRC proteins to significantly increased iron content in the temporal cortices of AD patients compared to CN individuals. Though the comparative nature of this postmortem study does not establish a direct cause-and-effect relationship, it has been previously demonstrated that there is a link between CAMKK2 loss and abnormal receptor-mediated *TF*-bound iron trafficking and iron homeostasis (Sabbir, 2018; Sabbir et al., 2020; Sabbir, 2020a). This link is reportedly supported by experiments involving CRISPR/Cas9-mediated *CAMKK2* deletion in transformed human cells (Sabbir, 2018; Sabbir et al., 2020), small interfering RNA-based knockdown in cultured primary rat dorsal root ganglion neurons (Sabbir, 2018), and *CAMKK2* knockout mouse models (Sabbir, 2020a). The significant reduction in temporal cortical CAMKK2 protein levels of both AD and PD patients compared to CN individuals aligns with previous studies suggesting a critical role for CAMKK2 in brain cells, specifically in neuronal function (Sakagami et al., 2000; Sakagami et al., 1998) and iron homeostasis (Sabbir, 2018; Sabbir, 2020a). Notably, the reduction in CAMKK2 was not influenced by age or PMI, indicating that the observed differences are likely disease-specific rather than age-related phenomena. One important question that arises from this study is whether the loss of CAMKK2 could be an initiating event in AD pathogenesis. This seems unlikely because no hallmark AD pathological features were observed in CAMKK2 knockout adult mouse brains, although some hippocampus-dependent long-term memory (LTM)-based behaviors, such as spatial memory formation, were affected, while others, such as contextual, trace fear, and passive avoidance, were not (Peters et al., 2003). Furthermore, although CAMKK2 is expressed in the cerebellum, as confirmed in this study, deletion of CAMKK2 in mice exhibited no apparent loss of cerebellar Purkinje cells, indicating that the loss of CAMKK2 in mice is not directly deleterious to neuronal survival (Peters et al., 2003). Additionally it is important to note that CAMKK2 activity can be independently modulated by several upstream kinases, including glycogen synthase kinase 3 (GSK3), cyclin-dependent kinase 5 (CDK5), and cAMP-activated protein kinase A (PKA) (Green et al., 2011; Racioppi and Means, 2012), which have been implicated in an array of neurodegenerative disorders, including AD (Llorens-Martin et al., 2014; Hooper et al., 2008; Kremer et al., 2011), PD (Golpich et al., 2015; Morales-Garcia et al., 2013; Choi and Koh, 2018) and Huntington’s disease (Cheung and Ip, 2012; Kawauchi, 2014). Previously, it was demonstrated that loss of CAMKK2 leads to cholinergic signaling-mediated abnormal Ca^2+^ release response, indicating a defective cholinergic pathway (Sabbir, 2020a). Cholinergic hypofunction and abnormal calcium signaling are both important parameters of AD pathogenesis (Alzheimer’s Association Calcium Hypothesis W, 2017; Sabbir et al., 2022), and this finding establishes a novel connection between these signaling events through CAMKK2. Therefore, it is likely that the loss of CAMKK2 may result from the dysregulation of one or more of these cell signaling pathways, which warrants further investigation. However, the novel finding that this loss is associated with significantly higher iron content and abnormally low TF/TFRC levels in the temporal cortex of AD patients compared to CN individuals provides important insights into AD pathogenesis.

The extent of CAMKK2 loss was found to be significantly lower in AD compared to PD, indicating disease-specific differences. There has been no previous report of CAMKK2 involvement in PD in the existing literature. However, a reduction in the activity of a Ca^2+^/CAM-dependent protein kinase subfamily protein, CAMK2, in the hippocampus has been associated with a dopaminergic neurotoxin-induced neurodegeneration mouse model exhibiting PD-like behaviors (Moriguchi et al., 2012). Furthermore, Bohush et al. reviewed the involvement of dysregulated Ca^2+^/CAM signaling in PD, highlighting the potential roles of multiple Ca^2+^/CAM kinases and downstream effectors (Bohush et al., 2021). Therefore, while the findings presented in this study suggest the involvement of CAMKK2 in PD, this needs further investigation. Another possibility that cannot be excluded is that up to 50% of patients with PD also develop AD pathologies (Aβ plaques and MAPT-containing neurofibrillary tangles) (Irwin et al., 2013), potentially contributing to the observed CAMKK2 reduction in the PD patient cohorts. In AD patients, the decrease in CAMKK2 was significant across different age groups, with a notable cluster of individuals aged 70–80 years showing very low CAMKK2 levels. This suggests a potential age-related susceptibility window where CAMKK2 reduction is particularly pronounced. The gender analysis indicated no significant difference in CAMKK2 levels between male and female AD patients, underscoring that the reduction is consistent across genders. Similarly, in PD patients, CAMKK2 levels were significantly reduced compared to CN individuals, and this reduction was not affected by age or PMI. This consistency across two different neurodegenerative diseases highlights CAMKK2’s potential role in broader neurodegenerative processes beyond AD.

This study also demonstrated significant reductions in TF and TFRC protein levels in the temporal cortex of both AD and PD patients compared to CN individuals. The reduction in TF and TFRC in AD correlated with a significantly increased iron content in the same tissue compared to CN individuals, suggesting a possible link to the iron dysregulation observed in AD cortices (Damulina et al., 2020; Spotorno et al., 2020; Smith et al., 1997; Kim et al., 2023; Ayton et al., 2020). Typically, a linear relationship between TF/TFRC content and iron content would be expected, meaning that if TF/TFRC levels decrease or increase, a similar decrease or increase in iron content would occur. For instance, one study involving 14 AD, 8 elderly CN, and 8 young CN found a significant increase in both TF and iron in the frontal cortex of AD patients compared to control groups (Loeffler et al., 1995). Conversely, another study using postmortem tissues consistently observed decreased TF in AD, particularly in the white matter of various cerebral cortical regions, while changes in iron and ferritin were inconsistent (Connor et al., 1992). However, this study observed an inverse relationship: the decrease in TF/TFRC significantly correlated with increased iron content in AD patients. This discrepancy could be due to brain region specific differences. From a mechanistic standpoint, this inverse relationship could occur if TF/TFRC is degraded after completing its cycle of iron transport or due to increased transcytosis of TF across the BBB. Notably, our previous study demonstrated increased transcytosis of TF across an *in vitro* BBB created using hypomorphic CAMKK2 (hemizygous deletion of CAMKK2 by CRISPR/Cas9) expressing EA.Hy926 cells, supporting this hypothesis (Sabbir et al., 2020). Furthermore, CAMKK2 deletion has been shown to affect TF post-translational modification, specifically phosphorylation, which was reflected by the loss of a negatively charged fraction of TF containing multiple serine, tyrosine, and threonine residues in different cell types, including neurons (Sabbir, 2018; Sabbir, 2020a). Phosphorylation of proteins acts as a signal determining protein function, trafficking, or fate (Bilbrough et al., 2022); however, TF phosphorylation has not been extensively studied by other researchers. Interestingly, TFRC is a target for phosphorylation by activated protein kinase C (PKC), which provides a signal for receptor internalization (May et al., 1985; May and Tyler, 1987), PKC is a calcium sensor (Jin et al., 2019) and both CAMKK2 and PKC are modulated by the effectors of CAM that regulates endocytosis (Kennedy et al., 2023). Therefore, it is possible that under reduced CAMKK2 conditions, alterations in the post-translational modifications of TF and TFRC may underlie the abnormal turnover of these proteins and iron dyshomeostasis. However, the involvement of non-TF/TFRC-mediated iron transport mechanisms cannot be ignored in the context of the observed increased iron content in the AD cortices. These possibilities require further investigation to establish the relationship between cortical TF/TFRC levels and iron content. Additionally, the correlation analysis revealed a complex relationship between TF levels and age in AD and PD patients. In AD patients, TF levels increased with age, while in PD patients, they decreased. This opposing trend suggests disease-specific mechanisms affecting TF regulation. For TFRC, no significant age-related trends were observed in any group, indicating that the reduction in TFRC is likely a disease-specific event rather than an age-related change.

One critical question that remains unaddressed is which brain cell types are involved in the observed dysregulation of the CAMKK-TF/TFRC-Iron dyshomeostasis in AD. This study could not address this question due to the limitations of performing immunohistochemical analysis on frozen postmortem tissue, which does not preserve the cellular details needed to pinpoint specific cell types with dysregulated CAMKK2 signaling. Interestingly, a recent study conducted immunohistochemical analysis on three serial sections from the frontal cortex (Brodmann areas 11 and 12) of three AD patients and three CN individuals, reporting no significant difference in the percentage of CAMKK2-positive neurons between the groups (Gaff et al., 2021). However, several factors suggest that this finding does not contradict the results of the present study. First, the study did not quantify CAMKK2 protein levels in individual brain cells but rather counted CAMKK2-positive cells - a method that introduces potential biases. Second, the small sample size (N = 3 per group) limits the statistical power, making it challenging to detect subtle differences. Additionally, neurons were identified based on morphological criteria, such as the pyramidal soma shape and presence of visible nuclei and nucleoli in brightfield images. This method introduces subjective bias, especially since the nucleolus (typically 0.5–3 microns) may not be visible in all neurons within a single 5 µm-thick section, depending on sectioning angle and cell size. Therefore, neuron identification solely based on morphology is suboptimal and could lead to misidentification. Another important limitation is that the study used an anti-CAMKK2 antibody (catalog number: sc-9629; Santa Cruz Biotechnology, Texas, USA) raised against a rat C-terminal epitope, which has not been validated for human proteins in paraffin-embedded brain tissue and negative controls. This raises concerns about antibody specificity and potential cross-reactivity that could compromise the accuracy of their results. Furthermore, immunohistochemical analysis, in general, is not ideal for quantifying protein level reductions between disease and control samples due to variable factors like chromogenic exposure, which can yield artifactual differences. To address these limitations comprehensively, future studies should leverage animal models and formaldehyde-fixed postmortem human brain tissues with cell-type-specific markers for neurons, astrocytes, and microglia. Such an approach will allow precise identification of CAMKK2 dysregulation within specific cell types and provide clearer insights into cell-specific iron homeostasis disruptions in AD. Meanwhile, existing literature provides some insights. According to the Human Protein Atlas’s single-cell RNA analysis, CAMKK2 is expressed in various brain cell types, including neurons, oligodendrocytes, astrocytes, and microglial cells (Karlsson et al., 2021). Several independent studies have also confirmed the expression of CAMKK2 in these cell types: neurons (Kokubo et al., 2009; Lee et al., 2022), oligodendrocytes (Cahoy et al., 2008), microglia (Huang et al., 2020; Cortes et al., 2017), astrocytes (Reyes-Ortiz et al., 2022), and endothelial cells (Sabbir et al., 2020; Reihill et al., 2007). Immunohistochemical analysis of TF and the iron storage protein ferritin, along with iron distribution in different brain regions from cognitively normal individuals aged 28–49 years and 60–90 years, revealed that oligodendrocytes contain much of the brain’s iron and iron-binding proteins (Connor et al., 1990). Aging is associated with altered cellular distribution of iron-binding proteins, specifically in glial cells (Connor et al., 1990). A recent study using iPSC-derived astrocytes and endothelial cells demonstrated that early-stage AD levels of Aβ disrupt iron transport signals secreted by astrocytes, affecting iron transport from endothelial cells (Baringer et al., 2023). This finding indicates the potential role of astrocytes and BBB endothelial cells in mediating brain iron homeostasis. Additionally, cortical neurons express high levels of both TF and TFRC (Abe et al., 2022). In the preclinical stage of AD, significant abnormal iron elevation has been observed in cortical neurons (Smith et al., 2010; Belaidi and Bush, 2016). Therefore, it is evident that CAMKK2-TF/TFRC-Iron dyshomeostasis may originate in any of these cell types. Future studies must systematically investigate this phenomenon to identify the specific cell types involved and understand the underlying mechanisms.

Overall findings in this study and our previous studies (Sabbir, 2018; Sabbir et al., 2020; Sabbir, 2020a) highlights CAMKK2 as a potential therapeutic target for modulating iron homeostasis in neurodegenerative diseases. Understanding the mechanisms through which CAMKK2 regulates TF and TFRC could lead to new therapeutic strategies aimed at restoring proper iron balance and mitigating the toxic effects of iron accumulation in the brain. In this context, it is important to note that CAMKK2 has emerged as a target for several neurological disorders (Kaiser et al., 2023). For example, its loss-of-function polymorphisms, missense mutations, and small nucleotide polymorphisms (SNPs) in humans are associated with behavioral disorders including anxiety, bipolar disorder, and schizophrenia (Luo et al., 2014; Erhardt et al., 2007; Scott et al., 2015). Furthermore, genetic deletion of Camkk2 in mice causes bipolar-like behaviors, which have been shown to be ameliorated by lithium treatment (Luo et al., 2014; Scott et al., 2015). Lithium increases CaMKK2 activity (Luo et al., 2014; Scott et al., 2015) by inhibiting its upstream regulator GSK3 (Chatterjee and Beaulieu, 2022). Interestingly, in mice, lithium treatment increases cortical iron, which is closely associated with neurodegeneration, cognitive loss, and parkinsonian features (Lei et al., 2017). Thus, there is a connection between the modulation of the GSK3-CAMKK2 pathway and iron homeostasis. While this connection is currently obscure, it is likely to become clearer as more research is conducted to unravel the details. Another important point to highlight is that reliance on postmortem tissue limits the ability to establish causality. Future studies using animal models and longitudinal human studies could provide deeper insights into the dynamic changes in CAMKK2, TF, and TFRC during disease progression. Additionally, exploring the molecular mechanisms linking CAMKK2 to iron homeostasis could reveal new targets for intervention. In conclusion, this study underscores the critical role of CAMKK2, TF, and TFRC in maintaining iron homeostasis in the brain and their significant alterations in AD and PD. These findings open new avenues for research into the pathophysiological mechanisms of neurodegenerative diseases and highlight potential targets for therapeutic intervention.

## Data Availability

The original contributions presented in the study are included in the article/Supplementary Material, further inquiries can be directed to the corresponding author.

## Supplementary material

SUPPLEMENTARY FIGURE S1 CAMKK2, TF, and TFRC protein levels in the temporal cortex tissues derived from male and female CN individuals and AD patient’s brains, postmortem. **(A–D)**: Immunoblots showing the relative abundance of CAMKK2 (A), TF **(B)**, TFRC **(C)**, and GAPDH **(D)** in temporal cortex tissues derived from human brains, postmortem. The sample identification numbers are provided on the top panel of each immunoblot. Males and females are highlighted in blue and red, respectively. CN and AD samples are color-coded blue and red, respectively.

SUPPLEMENTARY FIGURE S2 Correlation of CAMKK2 abundance with PMI or age of death variables in PD patients and the effect of age or gender on the relative abundance of TF and TFRC in AD patients. **(A, B)**: Scatter plots showing the abundance of CAMKK2 (%, Mean) in the temporal cortex combined with the PMI **(A)** and age of death **(B)** variables of the PD patients. The regression lines were drawn using GraphPad Prism software. **(C, D)**: Scatter plots showing temporal cortical abundance of TF and TFRC in male (n = 36) and female (n = 38) patients within the AD cohort (N = 74). P-values were determined by unpaired *t*-test. Ns = not significant. **(E)**: Scatter plot showing the TF and TFRC protein levels (%, mean from A) in the temporal cortex derived from different age groups of AD patients. P-values were determined by One-Way ANOVA followed by Dunnett’s Multiple Comparisons test.

SUPPLEMENTARY FIGURE S3 Correlation of TF and TFRC abundance in the temporal cortex with PMI variable in CN individuals, AD patients, and PD patients. **(A–F)**: Scatter plots showing the abundance of TF and TFRC (%, Mean) in the temporal cortex combined with the PMI in CN individuals **(A, D)**, respectively, AD patients **(B, E)**, respectively, and PD patients **(C, F)**, respectively. The regression lines were drawn using GraphPad Prism software. The solid red filled data point and the yellow arrow in the scatter plots **(A, D)** indicate the data point referring to the reference protein sample (#A21-006).

SUPPLEMENTARY TABLE S1 A cohort of prefrontal/temporal cortex tissue from 30 CN individuals of different age groups. BA10: Brodmann area 10, PMI: Postmortem Interval, RIN: RNA Integrity Number.
