# Supplementary figures and images for: Loss of calcium/calmodulin-dependent protein kinase kinase 2, transferrin, and transferrin receptor proteins in the temporal cortex of Alzheimer’s patients postmortem is associated with abnormal iron homeostasis: implications for patient survival
Source: Front Cell Dev Biol. 2024 Nov 28;12:1469751. doi: 10.3389/fcell.2024.1469751 (PMC11634808; doi:10.3389/fcell.2024.1469751)

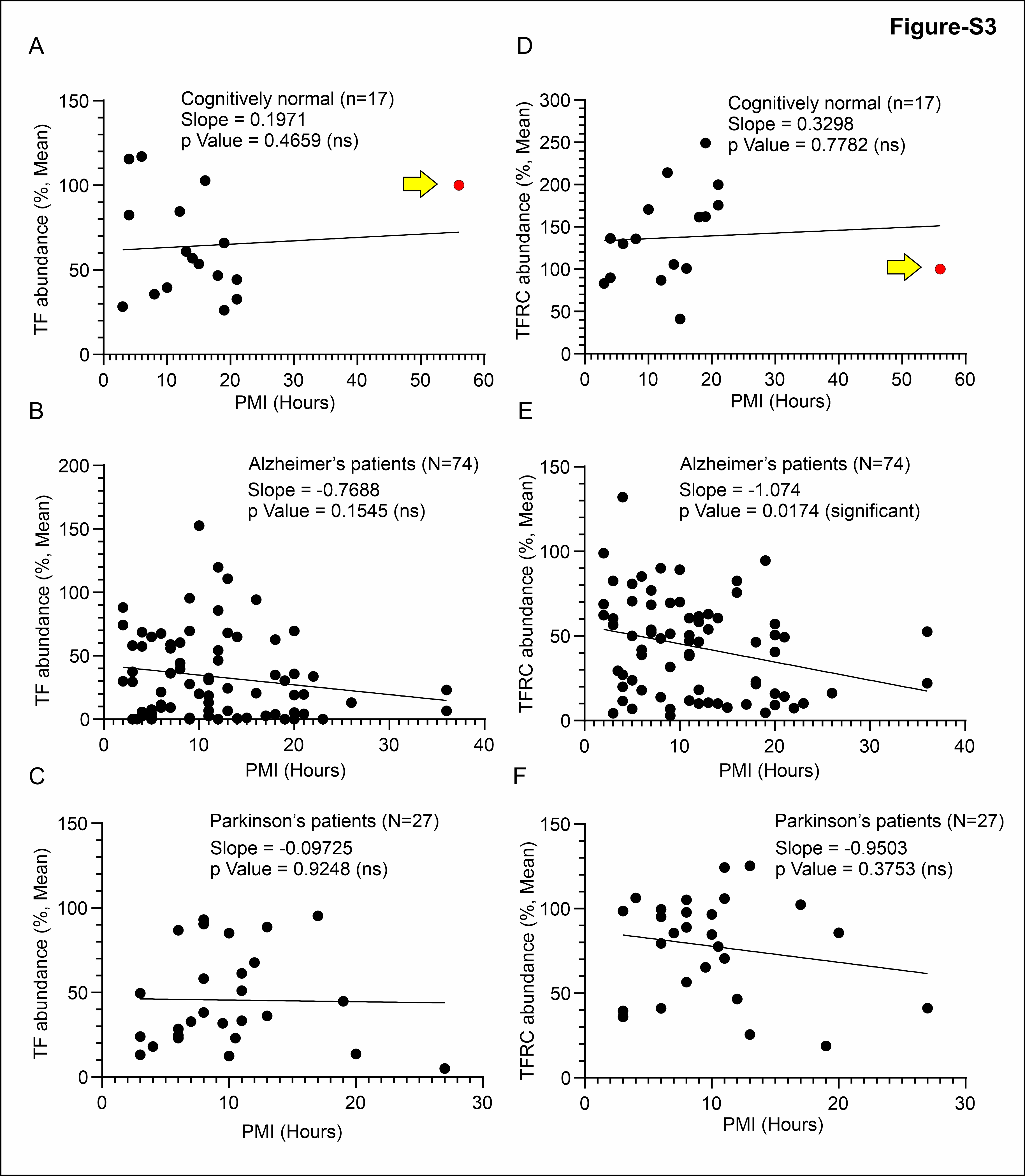

Supplement: Supplementary file 1 [file Image3.jpeg]

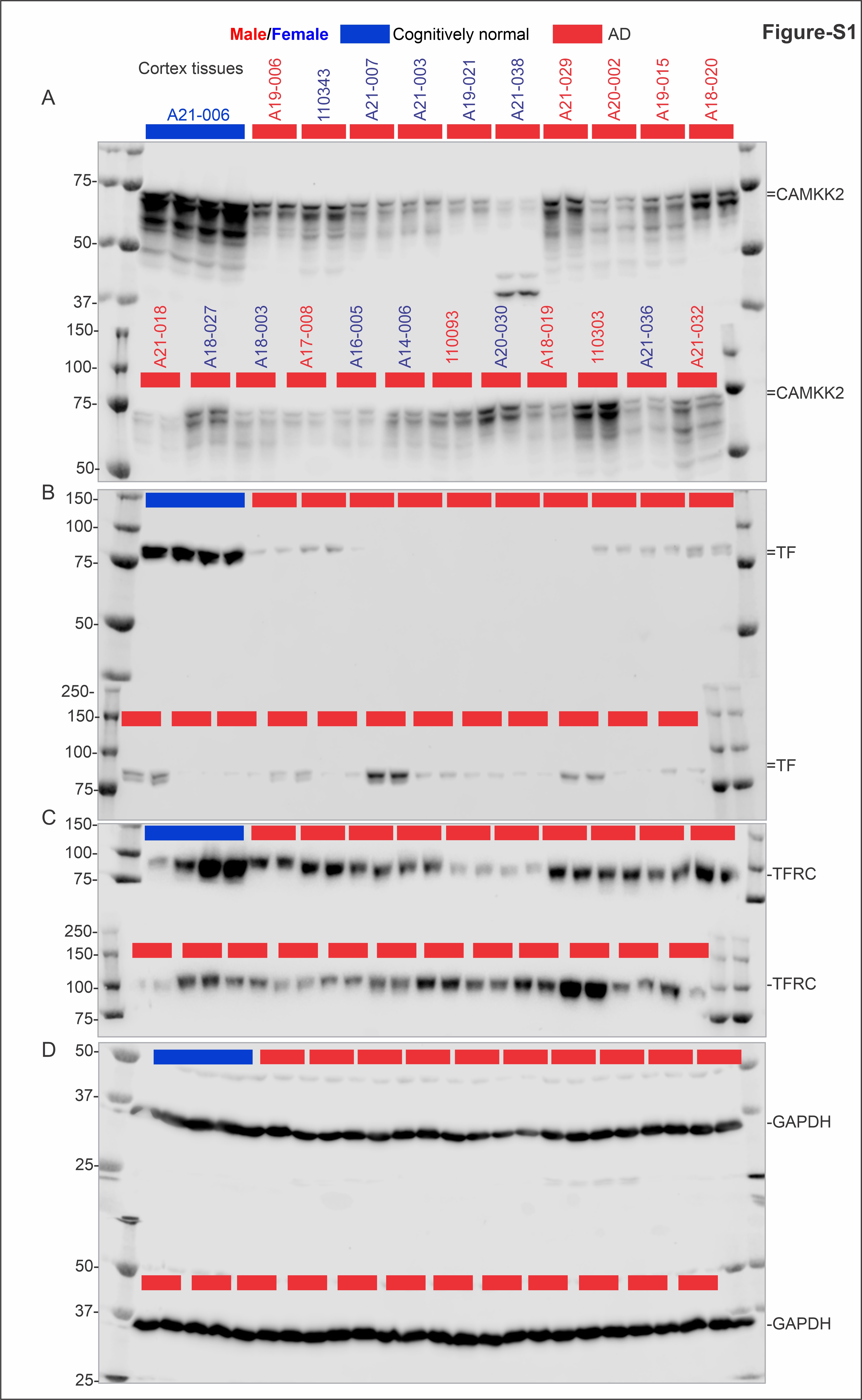

Supplement: Supplementary file 2 [file Image1.jpeg]

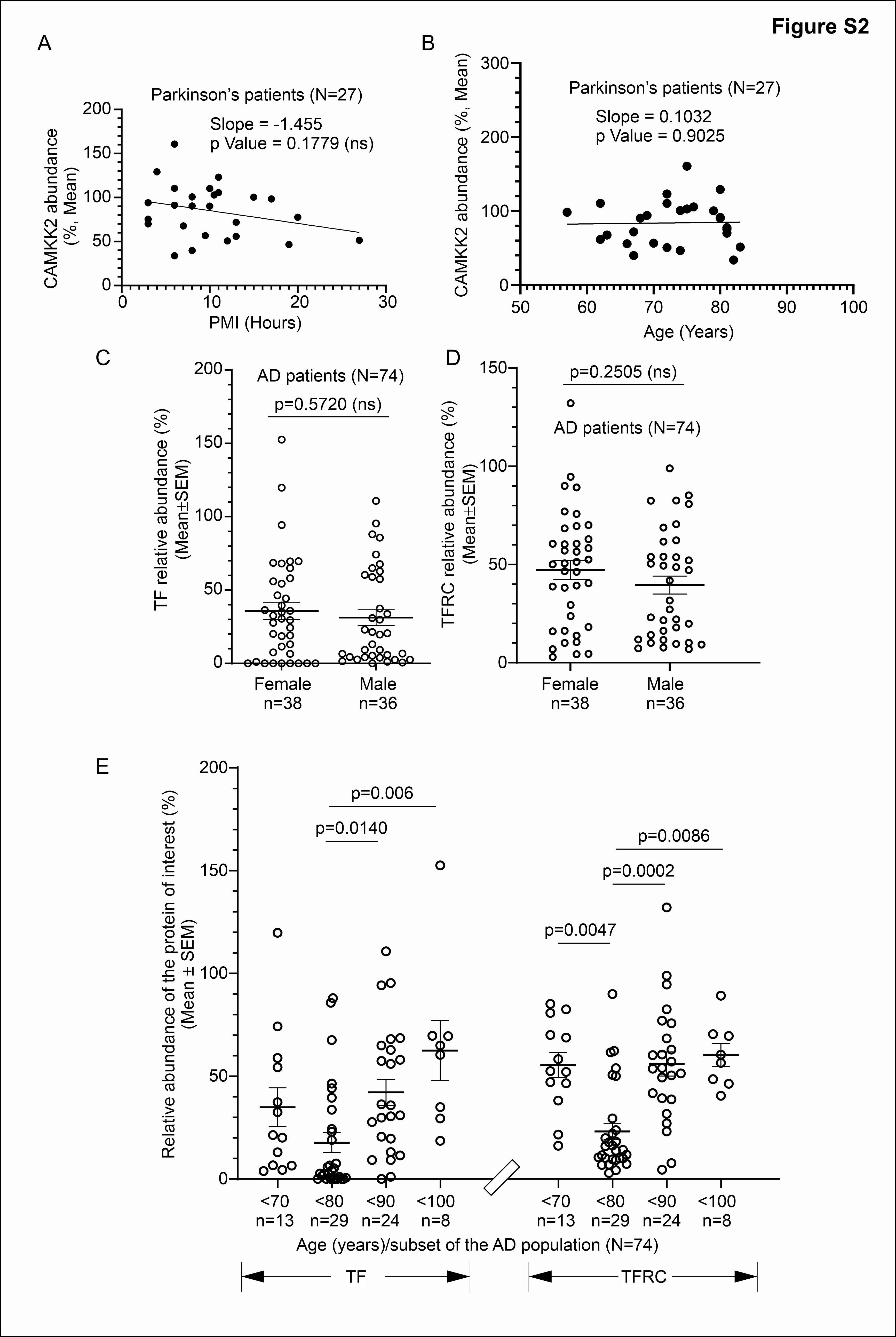

Supplement: Supplementary file 3 [file Image2.jpeg]
